# Identifying potential association on gene-disease network via dual hypergraph regularized least squares

**DOI:** 10.1186/s12864-021-07864-z

**Published:** 2021-08-09

**Authors:** Hongpeng Yang, Yijie Ding, Jijun Tang, Fei Guo

**Affiliations:** 1 School of Computer Science and Technology, College of Intelligence and Computing, Tianjin University, Tianjin, China; 2 Yangtze Delta Region Institute, University of Electronic Science and Technology of China, Quzhou, China; 3 Shenzhen Institute of Advanced Technology, Chinese Academy of Sciences, Shenzhen, China; 4 School of Computer Science and Engineering, Central South University, Changsha, China

**Keywords:** Gene-disease association network, Hypergraph learning, Dual Laplacian regularized least squares, Bipartite network, Multiple kernel learning

## Abstract

**Background:**

Identifying potential associations between genes and diseases via biomedical experiments must be the time-consuming and expensive research works. The computational technologies based on machine learning models have been widely utilized to explore genetic information related to complex diseases. Importantly, the gene-disease association detection can be defined as the link prediction problem in bipartite network. However, many existing methods do not utilize multiple sources of biological information; Additionally, they do not extract higher-order relationships among genes and diseases.

**Results:**

In this study, we propose a novel method called Dual Hypergraph Regularized Least Squares (DHRLS) with Centered Kernel Alignment-based Multiple Kernel Learning (CKA-MKL), in order to detect all potential gene-disease associations. First, we construct multiple kernels based on various biological data sources in gene and disease spaces respectively. After that, we use CAK-MKL to obtain the optimal kernels in the two spaces respectively. To specific, hypergraph can be employed to establish higher-order relationships. Finally, our DHRLS model is solved by the Alternating Least squares algorithm (ALSA), for predicting gene-disease associations.

**Conclusion:**

Comparing with many outstanding prediction tools, DHRLS achieves best performance on gene-disease associations network under two types of cross validation. To verify robustness, our proposed approach has excellent prediction performance on six real-world networks. Our research work can effectively discover potential disease-associated genes and provide guidance for the follow-up verification methods of complex diseases.

## Background

Identification of the association between disease and human gene has attracted more attention in the field of biomedicine, and has become an important research topic. A great deal of evidence shows that understanding genes related to diseases is of great help to prevent and treat diseases. However, identifying the relationship between disease and gene by biological experiments has to spend a long time and cost. Many computational models have been proposed to solve some similar biologically related problems. For example, in the fields of biology [1–3], pharmacy [4], and medicine [5, 6], machine learning methods help solve many analytical tasks.

In order to explore the relationship between gene and disease, a variety of algorithms have been proposed for association prediction. The typical machine learning methods [7–10] is to extract relevant features of known genetic data of each disease and train the model to determine which disease is related to those genes, so these algorithms are usually single-task algorithms for each disease. This model needs to be trained separately. Therefore, for a new disease or an existing disease with few known genes, due to the lack of known association data or the relevant information between various diseases, it is difficult to train the learning model. As a machine learning method, the matrix completion methods [11–13] can solve the above problem by calculating the similarity information and predicting the association between disease and gene, but the matrix completion method usually takes a long time to converge the local optimal solution. The other type is network-based model [14–17]. Li et al. [17] predicted the association by systematically embedding a heterogeneous network of genes and diseases into Graph Convolutional Network. This model usually divides genes and diseases into two heterogeneous networks. The edges in network represent the similarity between nodes. The model is based on the assumption that genes with high similarity are easily related to similar diseases. However, they are biased by the network topology, and it is necessary to rely on effective similarity information. It is not easy for these methods to integrate related sources of multiple genes and diseases.

Multiple Kernel Learning (MKL) is an important machine learning method, which can effectively combine multi-source information to improve the model effect, and is applied to many biological problems. For instance, Yu et al. [8] implemented one-class of Support Vector Machine while optimizing the linear combination of the gene nucleus and the MKL method. Ding et al. [18–21] proposed multiple information fusion models to identify drug-target and drug-side effect associations. Wang et al. [22] proposed a novel Multiple Kernel Support Vector Machine (MKSVM) classifier based on Hilbert Schmidt Independence Criterion to identify membrane proteins. Shen [23] and Ding et al. [24] proposed a MKSVM model to identify multi-label protein subcellular localization. Ding *et al* also employ fuzzy-besd model to predict DNA-binding proteins [25] and protein crystallization [26]. Zhang et al. [27] developed an ensemble predictive model of classifier chain to identify anti-inflammatory peptides.

LapRLS framework [28] is often used in various fields based on machine learning model, such as the prediction of Human Microbe-Disease Association [29] and the detection of human microRNA-disease association [30]. At the same time, Hypergraph learning [31–33] is becoming popular. Hypergraphs can represent more complex relationships among various objects. Bai et al. [34] introduced two end-to-end trainable operators to the family of graph neural networks, i.e., hypergraph convolution and hypergraph attention. Whilst hypergraph convolution defines the basic formulation of performing convolution on a hypergraph, hypergraph attention further enhances the capacity of representation learning by leveraging an attention module. Zhang et al. [35] developed a new self-attention based graph neural network called Hyper-SAGNN applicable to homogeneous and heterogeneous hypergraphs with variable hyperedge sizes. Ding et al. [36] predicted miRNAs-disease associations by a hypergraph regularized bipartite local model, which is based on hypergraph embedded Laplacian support vector machine.

Inspired by what is mentioned above, we propose a novel prediction method named Dual Graph Hypergraph Least Squares model (DHRLS) to predict gene-disease associations. Some computational models based on graph learning can effectively solve various network problems. In this paper, the gene-disease association detection can be defined as the link prediction problem in bipartite network [37–39]. Furthermore, two feature spaces are described by similarity information of multiple genes and diseases. Multiple kernel learning is also used to combine multiple informations linearly. Here, we use the Centered Kernel Alignment-based Multiple Kernel Learning (CKA-MKL) [40] to obtain weights of multiple kernels and then combine these kernels via optimal weights in two spaces, respectively. In addition, we also embed hypergraphs in graph regular terms to preserve high-order information of genes and diseases, using more complex information to improve prediction performance. To prove the effectiveness of our proposed method, six types of real networks and one gene-disease associations network are employed to test our predictive model. On the gene-disease associations dataset, our method has been compared with some methods under two types of cross-validation (CV). Comparing DHRLS with other state-of-the-art methods on predicting gene-disease associations, including CMF, GRMF and Spa-LapRLS, our model achieves the highest AUC and AUPR in 10-fold cross validation under CV1, but our model achieves lower AUC under CV2 compared with Spa-LapRLS. At the same time, DHRLS has excellent prediction performance on six benchmark datasets.

## Results

In order to better test the performance of our method, our proposed approach is verified on real gene-disease associations dataset under two types of cross validation. We also test the capability of DHRLS in predicting novel disease after confirming the excellent performance of our method based on cross validation. Furthermore, we employ benchmark datasets to evaluate our approach and compare it with other existing methods.

### Dataset

We download the dataset of gene-disease associations from [41] (http://cssb2.biology.gatech.edu/knowgene). Since the number of genes is too large and the information is insufficient, we remove some redundant gene data. Finally, our dataset contains 31,519 associations of 960 diseases and 3,118 genes, where 279 genes are associated with only one disease.

### Evaluation measurements

The 10-fold Cross Validation (CV) is usually used to verify the bipartite network detection. In order to compare the prediction performance with other methods under the same evaluation measurement, we will also use 10-fold CV for verification. At the same time, Area under the receiver operating characteristic curve (AUC) and Area Under the Precision-Recall curve (AUPR) as the major evaluation indicator, will also be applied to evaluate methods. There are two CV settings as follows:

**CV1**: Pair prediction. All gene-disease associations are randomly divided into test set and training set, and the associations in the test set are removed.

**CV2**: Disease prediction. All diseases are randomly divided into test set and training set, and all associations of diseases in the test set are removed.

### Parameter settings

In our study, DHRLS has some parameters *λ*_*d*_,*λ*_*g*_,*β*, *k* and number of iterations. In the parameter selection, we consider all combinations of following values: number of *k*-Nearest Neighbor is from 10 to 100 (with step 10); number of iterations is {1,2,...,15}; {2^−5^,...,2^0^,...,2^5^} for *λ*_*d*_ and *λ*_*g*_; *β*=1.

**Fig. 1:**
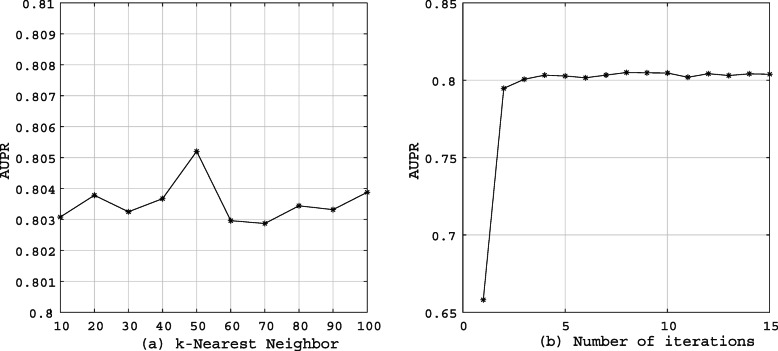
Different AUPR values under different *k*-Nearest Neighbor and iterations

Figure 1 shows the results of our model obtained under different iteration times and *k* values. For the number of *k*-Nearest Neighbor, we select the optimal *k* under the highest AUPR value and can clearly find that AURP reaches its peak when *k*=50. For the number of iterations, it basically converges at the four times. In order to train the model more fully, we finally choose the number of iterations to be 10.

**Fig. 2:**
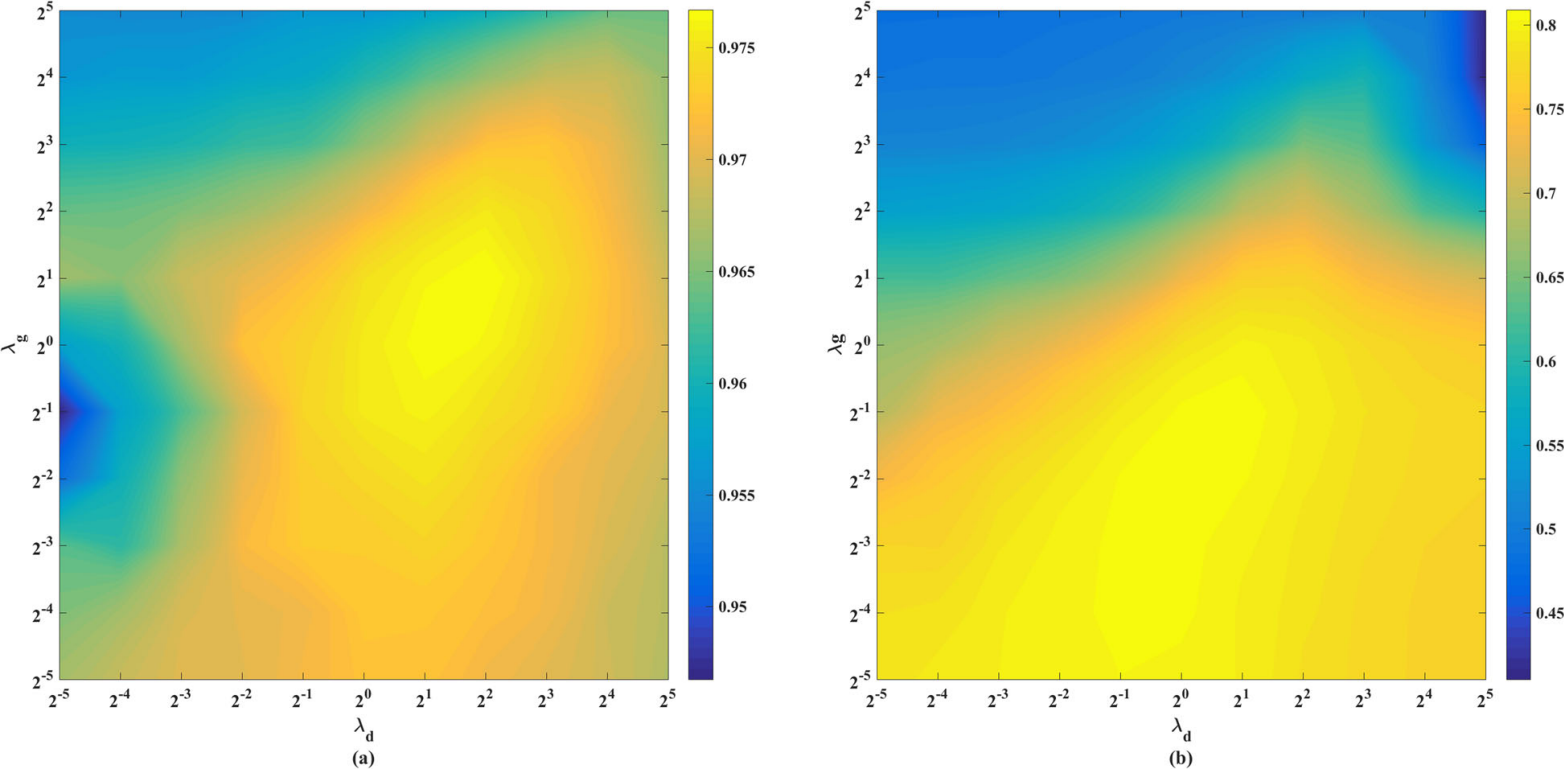
The AUC (a) and AUPR (b) of models with different *λ*_*d*_ and *λ*_*g*_ under CV1. *λ*_*d*_ (horizontal axis) and *λ*_*g*_ (vertical axis) are set from 2^−5^ to 2^5^ with step 2^1^. The yellow color is the higher value, and blue color is the lower value

Figure 2 shows the results of AUC and AUPR in grid search for parameters *λ*_*d*_ and *λ*_*g*_. The optimal *λ*^*d*^ and *λ*^*s*^ are also selected under highest AUPR value. In this study, the optimal parameters of Hypergraph Laplace regular terms are obtained on *λ*_*d*_=1 and *λ*_*g*_=0.25. Under this parameter selection, the AUC value is relatively high.

### Evaluation on gene-disease association data

#### Performance analysis

**Fig. 3:**
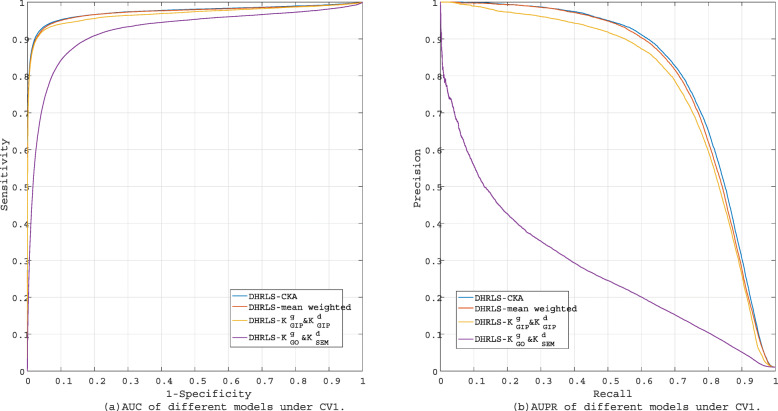
The performance of different models under CV1

We evaluate the different performance of CKA-MKL, mean weighted-based MKL and single kernel $\left (\mathbf {K}^{d}_{SEM} \& \mathbf {K}^{g}_{GO} \text {and} \mathbf {K}^{d}_{GIP} \& \mathbf {K}^{g}_{GIP}\right)$. The testing results are shown in Table 1 and Fig. 3.

**Table 1:** The performance of different models under CV1

Model	AUC	AUPR
CKA-MKL + DHRLS	**0.9742**	**0.8092**
mean weighted + DHRLS	0.9703	0.8006
$\mathbf {K}^{d}_{GIP} \& \mathbf {K}^{g}_{GIP}$ + DHRLS	0.9554	0.7377
$\mathbf {K}^{d}_{SEM} \& \mathbf {K}^{g}_{GO}$ + DHRLS	0.9154	0.2827

Obviously, the model of CKA-MKL on DHRLS obtains the best performance with AUC of 0.9742 and AUPR of 0.8092. Comparing with mean weighted on DHRLS, AUPR and AUC are increased by 0.0086 and 0.0039. This means that CKA combines multi-kernel information more effectively than simple average combination. What’s more, DHRLS with single kernel $\left (\mathbf {K}^{d}_{SEM} \& \mathbf {K}^{g}_{GO}\right)$ obtains lower performance than the model with GIP kernel. Therefore, GIP is an effective method to calculate the kernel matrix. By comparing the results of single kernel and multi-kernel models, combining multiple information is an effective method to improve the prediction effect of the model.

**Fig. 4:**
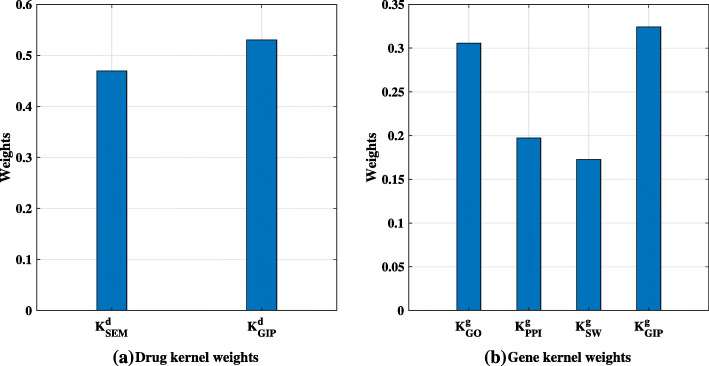
The kernel weights of drug and gene space respectively

Furthermore, Fig. 4 shows the weights of each kernel matrix in the gene space and disease space. The weight of the kernel indicates the degree of contribution of the corresponding kernel matrix. Comparing the weights in the gene and disease spaces, the GIP kernel has a higher weight in both spaces, which is consistent with the results in Table 1. In gene space, except for GIP kernel, the kernel weight of $\mathbf {K}^{g}_{GO}$ is higher than $\mathbf {K}^{g}_{PPI}$ and $\mathbf {K}^{g}_{SW}$. This means that $\mathbf {K}^{g}_{GO}$’s contribution to the overall is better than the other two kernel matrices.

#### Comparison to existing predictors

**Fig. 5:**
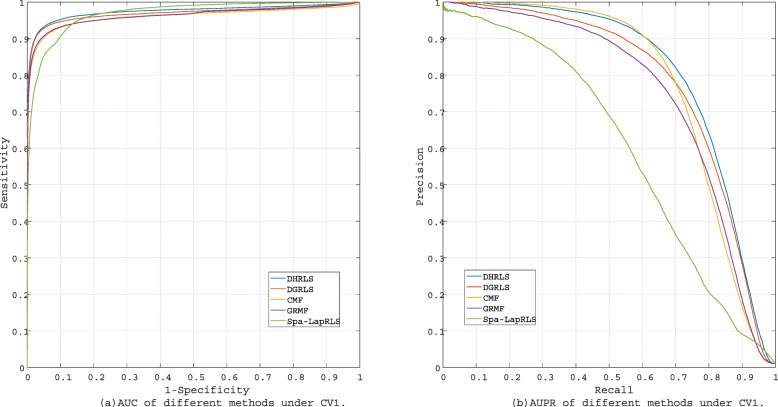
The performance of different methods under CV1

Many excellent methods have been proposed to predict the bipartite network link, including Spa-LapRLS [30], GRMF [42] and CMF [43]. Our method is compared to the existing methods and DGRLS under CV1 and CV2, respectively. Under CV1, the results are shown in Table 2 and Fig. 5. Our method achieves the best AUC (0.9742) and AUPR (0.8092). For AUC, DHRLS is not much different from DGRLS and Spa-LapRLS, which is about 0.01 higher than GRMF and CMF. As for AUPR, DHRLS achieves better performance than other methods. Comparing the results of DHRLS and DGRLS, it can be seen that the hypergraph-based model is better than the normal graph model, which shows that the high-level graph information constructed by the hypergraph is helpful for the predict performance. This is related to the ability of hypergraph to effectively find similar information between nodes. At the same time, the methods based on LapRLS (DHRLS, DGRLS and Spa-LapRLS) are higher than those based on matrix factorization (GRMF and CMF), indicating that the model framework of LapRLS has more advantages in the prediction of gene-disease associations.

**Table 2:** The performance of different methods under CV1

Method	AUC	AUPR
DHRLS	**0.9742**	**0.8092**
DGRLS	0.9700	0.7842
Spa-LapRLS	0.9704	0.6222
GRMF	0.9609	0.7521
CMF	0.9594	0.7823

**Fig. 6:**
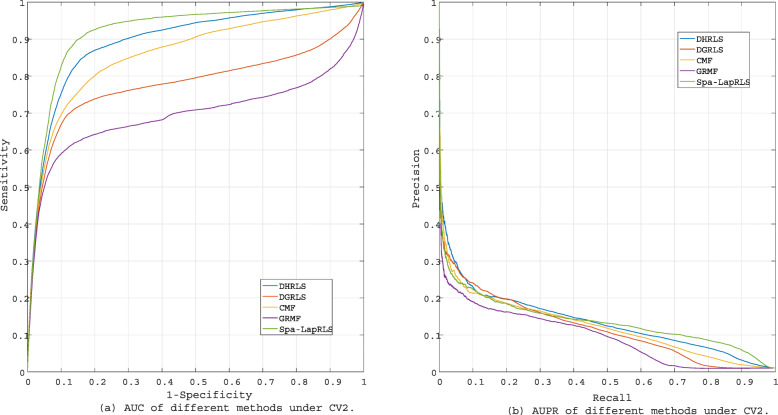
The performance of different methods under CV2

In order to test the performance of our method detecting new diseases, the associations for new diseases (CV2) are not observed in the training set. Table 3 and Fig. 6 show the results of CV2. Under CV2, our method obtains best AUPR (0.1413). However, the performance of our model on AUC (0.8987) is secondary best, which is about 0.02 lower than that of Spa-LapRLS.

**Table 3:** The performance of different methods under CV2

Method	AUC	AUPR
DHRLS	0.8987	**0.1413**
DGRLS	0.7887	0.1233
Spa-LapRLS	**0.9199**	0.1402
GRMF	0.7240	0.1013
CMF	0.8640	0.1256

Comparing the results of DGRLS and DHRLS under CV1 and CV2, we clearly find that utilizing hypergraph to establish higher-order relationships greatly improves the predictive ability of the model.

#### Case study

Our model can predict genes associated with new diseases. Here, we use DHRLS to rank the predicted values of genes related to new diseases in descending order. The higher the ranking, the more likely it is to interact. We set the value of a disease in the correlation matrix to 0 as a new disease. One example is Lung Diseases. We intercepted the top 50 predicted genes and 40 (80%) known related genes in the predicted results. All predicted ranking results are shown in Table 4.

**Table 4:** Predicted top 50 genes for Lung Diseases by our method

Rank	Gene	Confirm	Rank	Gene	Confirm
1	DQB1_HUMAN	Y	26	PBX2_HUMAN	Y
2	DQA1_HUMAN	Y	27	BRD2_HUMAN	Y
3	IFNG_HUMAN	N	28	TGFB1_HUMAN	N
4	DQA2_HUMAN	Y	29	CFTR_HUMAN	Y
5	ACE_HUMAN	Y	30	PAFA_HUMAN	Y
6	TNFA_HUMAN	Y	31	TAP2_HUMAN	N
7	DPB1_HUMAN	Y	32	UBP38_HUMAN	Y
8	CTLA4_HUMAN	N	33	MUC7_HUMAN	Y
9	ADA33_HUMAN	Y	34	DPP10_HUMAN	Y
10	NOTC4_HUMAN	Y	35	CH3L1_HUMAN	Y
11	PDE4D_HUMAN	Y	36	IL6RA_HUMAN	Y
12	IL13_HUMAN	N	37	RNBP6_HUMAN	Y
13	DRA_HUMAN	Y	38	CHIT1_HUMAN	Y
14	SMAD3_HUMAN	Y	39	ELF3_HUMAN	Y
15	IL4_HUMAN	N	40	ORML3_HUMAN	Y
16	DPA1_HUMAN	Y	41	S2546_HUMAN	Y
17	SUOX_HUMAN	Y	42	CDK2_HUMAN	Y
18	IKZF4_HUMAN	Y	43	IL2RB_HUMAN	Y
19	EMSY_HUMAN	Y	44	IL33_HUMAN	Y
20	IL18R_HUMAN	Y	45	DOA_HUMAN	Y
21	ILRL1_HUMAN	Y	46	X5CF87_HUMAN	Y
22	ZNT8_HUMAN	Y	47	TBX21_HUMAN	N
23	IL10_HUMAN	N	48	IL12A_HUMAN	N
24	CRUM1_HUMAN	Y	49	2B1G_HUMAN	N
25	TSLP_HUMAN	Y	50	PSPB_HUMAN	Y

### Evaluation on six benchmark datasets

To test the performance of our proposed method, we consider six real-world networks: (i) G-protein coupled receptors (GPC Receptors): the biological network of drugs binding GPC receptors; (ii)Ion channels: the biological network of drugs binding ion channel proteins; (iii) Enzymes: the biological network of drugs binding enzyme proteins; (iv) Southern Women (referred here as “SW”): the social relations network of women and events; (v) Drug-target: the chemical network of drug-target interaction; (vi) Country-organization (referred here as “CO”): the network of organization most related to the country. Detailed information about six datasets is described in Table 5.

**Table 5:** Statistics of six real-world networks

Network	|**V**|	|**W**|	|**E**|	LD	AD	LAD	RAD
GPC	95	223	635	0.0300	2.00	6.68	2.85
Enzymes	664	445	2926	0.0099	2.64	4.41	6.58
Ionchannel	210	204	1476	0.0345	3.57	7.03	7.24
Drug-target	200	150	454	0.0151	1.30	2.27	3.03
SW	18	14	89	0.3532	2.78	4.94	6.36
CO	144	151	12170	0.5597	41.25	84.51	80.60

|**V**|,|**W**| denote the number of two types of nodes respectively; |**E**| is the number of edges; LD, AD, LAD, and RAD are the link density, the average degree, the left average degree, the right average degree.

Since there is only the data of interaction matrix of binary network, in order not to introduce additional data, we directly use the GIP kernel extracted from the interaction matrix as the kernel matrix for each real-world network. The kernel is defined as follows:

1a $$\begin{array}{*{20}l} \mathbf{K}^{*}_{1}(i,j) &= \exp \left(-\gamma_{1}||\mathbf{Y}_{i}-\mathbf{Y}_{j}||^{2}\right) \end{array} $$

1b $$\begin{array}{*{20}l} \mathbf{K}^{*}_{2}(m,n) &= \exp \left(-\gamma_{2}||\mathbf{Y}^{T}_{m}-\mathbf{Y}^{T}_{n}||^{2}\right) \end{array} $$

where **Y** is the train set of binary network, and **Y**_*i*_ is the vector of associations.

We test our method on above six datasets and compare results with other methods [44]. Wang et al. [44] proposed a framework, called Similarity Regularized Nonnegative Matrix Factorization (SRNMF), for link prediction in bipartite networks by combining the similarity based structure and the latent feature model from a new perspective. Tables 6 and 7 show the comparison of precision and AUC for six real-world networks. DHRLS performs better than other methods on Enzymes and Ionchannel networks, and values of our precision and AUC are higher than others. For GPC and Drug-target networks, the precision is same, but AUC is slightly higher. This directly indicates the clear performance advantage of our approach in real-world binary networks.

**Table 6:** Precision by different methods on six real networks

Method	GPC	Enzymes	Ionchannel	Drug-target	SW	CO
DHRLS	0.43	0.73	0.74	0.75	0.26	0.94
SRNMF-CN	0.41	0.69	0.69	0.74	0.20	0.94
SRNMF-AA	0.43	0.69	0.69	0.74	0.22	0.92
SRNMF-JC	0.43	0.69	0.69	0.74	0.23	0.93
SRNMF-CAA	0.42	0.69	0.69	0.74	0.20	0.93
SRNMF-CJC	0.42	0.69	0.69	0.73	0.22	0.94

Comparison refers to the reference [44].

**Table 7:** AUC by different methods on six real networks

Method	GPC	Enzymes	Ionchannel	Drug-target	SW	CO
DHRLS	0.89	0.96	0.97	0.98	0.85	1.00
SRNMF-CN	0.84	0.88	0.94	0.93	0.83	1.00
SRNMF-AA	0.83	0.88	0.94	0.93	0.82	1.00
SRNMF-JC	0.83	0.88	0.93	0.92	0.85	1.00
SRNMF-CAA	0.83	0.87	0.94	0.92	0.82	1.00
SRNMF-CJC	0.83	0.87	0.95	0.93	0.80	1.00

Comparison refers to the reference [44].

## Discussion

We developed the model DHRLS for the gene-disease association prediction. In order to evaluate our model, we test not only on real gene-disease associations dataset, but also on some benchmark datasets. By comparing the results of single-kernel model and multi-kernel model, MKL can effectively combine multi-kernel information to improve the predictive ability of the model. By adjusting different kernel weights, different kernel matrices can express different levels of information. However, MKL needs to be applied to samples with multiple feature information, and the application effect is not obvious for problems with fewer features. The comparison of DHRLS and DGRLS can illustrate the effectiveness of hypergraph. After adding the hypergraph, the result of the model is obviously improved, which is caused by the characteristics of the hypergraph. Hypergraph uses high-order information between nodes, that is, a hyperedge can connect more than two nodes, which can better indicate the degree of similarity between nodes. Comparing DHRLS with other state-of-the-art methods on predicting gene-disease associations, including CMF, GRMF and Spa-LapRLS, our model achieves the highest AUC and AUPR in 10-fold cross validation under CV1, but our model achieves lower AUC under CV2 compared with Spa-LapRLS. At the same time, DHRLS has excellent prediction performance on six benchmark datasets.

Nevertheless, our model still has some flaws.First of all, the model contains a large number of matrix operations and optimization problems, and lacks a certain degree of simplicity. Secondly, we need to calculate the multi-kernel information of the sample. Therefore, we cannot achieve predictions for samples without features. At present, most of the computational methods are developed to predict the associations of gene-disease, and there is still a great possibility to improve the prediction performance. For example, hypergraph can be considered in the graph based method. In the future, for optimizing the model and improving the prediction performance, we can add some data preprocessing and calculate simplification on the basis of DHRLS, as well as better method to build hypergraph.

## Conclusion

In summary, we propose a Dual Hypergraph Regularized Least Squares (DHRLS) based on CKA-MKL algorithm, for the gene-disease association prediction. We use multiple kernels to describe gene and disease spaces. The weights of these kernels are obtained by CKA-MKL and used to combine kernels. We use hypergraph to describe more complex information to improve our prediction. Our purpose is to establish an accurate and effective prediction model of gene-disease association based on the existing data of gene-disease associations, and provide guidance for the follow-up verification methods of complex diseases.

## Methods

**Fig. 7:**
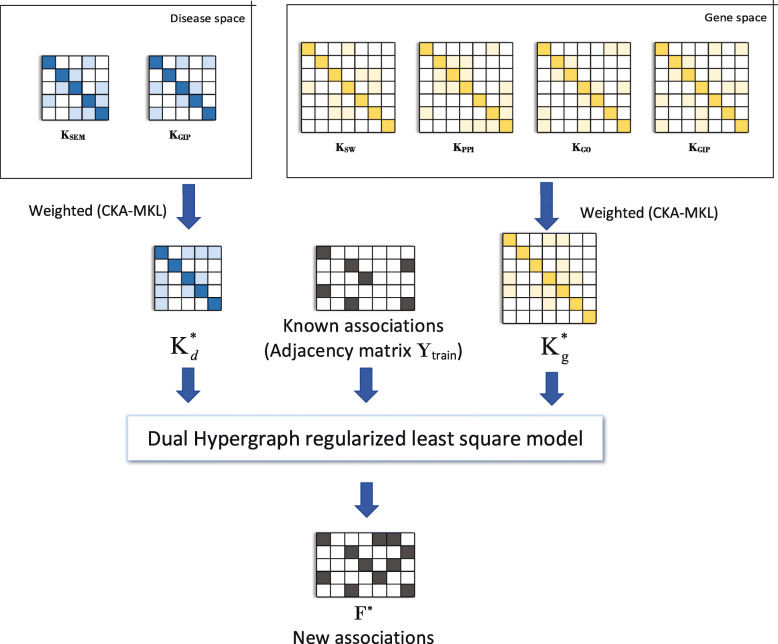
The overview of our proposed method

In this study, we first use two disease kernels and four gene kernels to reveal potential associations of genes and diseases. Then, the MKL method CKA is used to combine above kernels into one disease kernel and one gene kernel. Finally, we use Dual Hypergraph Regularized Least Squares to identify gene-disease associations. Figure 7 show the flowchart of our method DHRLS.

### Problem definition

The prediction of gene-disease associations can be regarded as a recommendation system. Given *n* diseases *D*={*d*_1_,*d*_2_,,...,*d*_*n*_}, *m* genes *S*={*g*_1_,*g*_2_,,...,*g*_*m*_} and gene-disease associations. The association between gene and disease items can be expressed as an adjacent matrix **Y**∈**R**^*n*×*m*^. The element of adjacent matrix **Y** is the relationship between genes and diseases. If disease *d*_*j*_(1≤*j*≤*m*) is associated with gene *g*_*i*_(1≤*i*≤*n*), the value of **Y**_*i*,*j*_ is set as 1, otherwise it is 0. Genes, diseases, and their associations are formulated as a bipartite network.

### Related work

LapRLS framework [28] is often used in various fields based on machine learning model, such as the prediction of Human Microbe-Disease Association [29] and the detection of human microRNA-disease association [30]. At the same time, Hypergraph learning [31–33] is becoming popular. Hypergraphs can represent more complex relationships among various objects.

The Laplacian Regularized Least Squares (LapRLS) model [45] based on graph regularization is employed to predict potential associations in a bipartite network. The functions of model can be defined as follows:

2 $$ \begin{aligned} &{}\mathbf{F}^{*}_{a}=\arg\min{J}(\mathbf{F}_{a}) = ||\mathbf{Y}_{train}-\mathbf{F}_{a}||^{2}_{F}+\lambda_{a}tr\left(\mathbf{F}^{T}_{a}\mathbf{L}_{a}\mathbf{F}_{a}\right)\\ &{}\mathbf{F}^{*}_{b}=\arg \min {J}(\mathbf{F}_{b}) = ||\mathbf{Y}_{train}-\mathbf{F}_{b}||^{2}_{F}+\lambda_{b}tr\left(\mathbf{F}^{T}_{b}\mathbf{L}_{b}\mathbf{F}_{b}\right) \end{aligned}  $$

where $\mathbf {F}^{*}_{a}=\mathbf {K}_{a}\pmb {\alpha }^{*}_{a}, \mathbf {F}^{*}_{b}=\mathbf {K}_{b}\pmb {\alpha }^{*}_{b}$, and $\mathbf {F}_{a}, \pmb {\alpha }^{*}_{a}, \mathbf {F}^{T}_{b}, {\pmb {\alpha }^{*}_{b}}^{T}, \mathbf {Y}_{train} \in \mathbf {R}^{n\times m}$. **K**_*a*_∈**R**^*n*×*n*^ and **K**_*b*_∈**R**^*m*×*m*^ are kernels in two feature space, separately.

**L**_*a*_∈**R**^*n*×*n*^ and **L**_*b*_∈**R**^*m*×*m*^ are the normalized Laplacian matrices as follows:

3 $$ \begin{aligned} \mathbf{L}_{a}=\mathbf{D}^{-1/2}_{a}\Delta_{a}\mathbf{D}^{1/2}_{a},&\Delta_{a}=\mathbf{D}_{a}-\mathbf{K}_{a}\\ \mathbf{L}_{b}=\mathbf{D}^{-1/2}_{b}\Delta_{b}\mathbf{D}^{1/2}_{b},&\Delta_{b}=\mathbf{D}_{b}-\mathbf{K}_{b} \end{aligned}  $$

where **D**_*a*_ and **D**_*b*_ are diagonal matrices, $\mathbf {D}_{a}(k,k)=\sum _{l=1}^{n}\mathbf {K}_{a}(k,l), \mathbf {D}_{b}(k,k)=\sum _{l=1}^{m}\mathbf {K}_{b}(k,l)$

The variables *α*_*a*_ and $\pmb {\alpha }^{*}_{b}$ of LapRLS can be solved as follows:

4 $$ \begin{aligned} \pmb{\alpha}^{*}_{a}&=\left(\mathbf{K}_{a}+\lambda_{a}\mathbf{L}_{a}\mathbf{K}_{a}\right)^{-1}\mathbf{Y}_{train}\\ \pmb{\alpha}^{*}_{b}&=(\mathbf{K}_{b}+\lambda_{b}\mathbf{L}_{b}\mathbf{K}_{b})^{-1}(\mathbf{Y}_{train})^{T} \end{aligned}  $$

And $\mathbf {F}^{*}_{a}$ and $\mathbf {F}^{*}_{b}$ can be calculated as follows:

5 $$ \begin{aligned} \mathbf{F}^{*}_{a}&=\mathbf{K}_{a}(\mathbf{K}_{a}+\lambda_{a}\mathbf{L}_{a}\mathbf{K}_{a})^{-1}\mathbf{Y}_{train}\\ \mathbf{F}^{*}_{b}&=\mathbf{K}_{b}\left(\mathbf{K}_{b}+\lambda_{b}\mathbf{L}_{b}\mathbf{K}_{b}\right)^{-1}\left(\mathbf{Y}_{train}\right)^{T} \end{aligned}  $$

The predictions from two feature spaces are combined into:

6 $$ \mathbf{F}^{*}=\frac{\mathbf{F}^{*}_{a}+(\mathbf{F}^{*}_{b})^{T}}{2}  $$

### Feature extraction

To improve effectiveness of detecting gene-disease associations, We use two and four types of similarity for disease and gene separately. In our work, we constructed the multiple kernels of diseases and genes to represent the feature sets. Table 8 summarizes whole kernels, including two feature spaces.

**Table 8:** Summary of kernels in two feature spaces

Space	Kernel	Description
Disease	$\mathbf {K}^{d}_{SEM}$	Semantic similarity for disease
	$\mathbf {K}^{d}_{GIP}$	Gaussian interaction profile for disease
Gene	$\mathbf {K}^{g}_{GO}$	Functional information of gene
	$\mathbf {K}^{g}_{PPI}$	Protein-protein interactions(PPIs) network of gene
	$\mathbf {K}^{g}_{SW}$	Sequence information of gene
	$\mathbf {K}^{g}_{GIP}$	Gaussian interaction profile for gene

#### Disease space

We calculate two classes of disease kernels, including semantic similarity kernel and Gaussian Interaction Profile (GIP) kernel (for disease).

**a) Semantic similarity** The disease semantic similarity kernel is calculated by the relative positions in the MeSH [46] disease. Directed Acyclic Graph (DAG) [47] can describe disease *d*_*i*_ as a node. A disease *d*_*i*_ can be described as a node in DAG and denoted as ${DAG}_{d_{i}} = (d_{i}, T_{d_{i}}, E_{d_{i}}) $, where $T_{d_{i}}$ is the set of all ancestor nodes of *d*_*i*_ including node *d*_*i*_ itself and $E_{d_{i}}$ is the set of corresponding links. A semantic score of each disease $ t \in T_{d_{i}}$ can be calculated as follows:

7 $$  D_{d_{i}}(t) \left\{\begin{array}{ll} 1& \text{if~} t =d_{i} \\ \max \{\Delta \ast D_{d_{i}}(t^{'})|t^{'} \in children \ of \ t \}& \text{if~} t \not= d_{i} \end{array}\right.  $$

where *Δ* is the semantic contribution factor, which is set to 0.5 in this paper.

Then, the semantic score of disease *d*_*i*_ can be calculated as follows:

8 $$  DV(d_{i})={\sum\nolimits}_{t \in T_{d_{i}}}D_{d_{i}}(t)  $$

So, the disease semantic similarity kernel $K^{d}_{SEM} \in \mathbf {R}^{n\times n}$ is calculated as follows:

9 $$  K^{d}_{SEM}\left(d_{i},d_{j}\right)= \frac{{\sum\nolimits}_{t \in T_{d_{i}} \bigcap T_{d_{j}}}\left(D_{d_{i}}(t)+D_{d_{j}}(t)\right)} {DV(d_{i})+DV(d_{j})}  $$

**b) GIP kernel similarity** The similarity between diseases can also be calculated by GIP. Given two diseases *d*_*i*_ and *d*_*j*_(*i*,*j*=1,2,...,*n*), the GIP kernel can be calculated as follows:

10 $$  K^{d}_{GIP}\left(d_{i},d_{j}\right)= \exp \left(-\gamma_{d}||\mathbf{Y}_{d_{i}}-\mathbf{Y}_{d_{j}}||^{2}\right)  $$

where $\mathbf {Y}_{d_{i}}$ and $\mathbf {Y}_{d_{j}}$ are the information of associations for vector disease *d*_*i*_ and *d*_*j*_. *γ*_*d*_ (set as 0.5) is the bandwidth of GIP kernel.

#### Gene space

Four types of gene kernels, including Gene Ontology (GO) [48] similarity, Protein-protein interactions (PPIs) network similarity, sequence similarity kernel and GIP kernel (for gene) are utilized to represent the relationship between genes.

**a) GO similarity** The information of GO is obtained through DAVID [49]. GO similarity $\left (K^{g}_{GO} \in \mathbf {R}^{m \times m}\right)$ is the overlap of GO annotations on two genes, and we simply use GOSemSim [50] to get it. We consider one option of GO: cellular component (CC) to represent gene functional annotation.

**b) PPIs similarity** We download the protein-protein interactions network from previous research [41] and select the sub-networks related to our genes. Give the topological feature vectors *p*_*i*_ and *p*_*j*_ of two genes in the PPIs network. The Cosine similarity of PPIs network can be calculated as follows:

11 $$  K^{g}_{PPI}\left(p_{i},p_{j}\right)= \frac{p_{i} \cdot p_{j}}{||p_{i}||||p_{j}||}  $$

**c) Sequence similarity** We use the normalized Smith Waterman (SW) score [51] to measure the sequence similarity between the two gene sequences, which is calculated as follows:

12 $$  K^{g}_{SW}\left(g_{i},g_{j}\right)= \frac{SW\left(S_{g_{i}},S_{g_{j}}\right)}{\sqrt{SW\left(S_{g_{i}},S_{g_{i}}\right)} \sqrt{SW\left(S_{g_{j}},S_{g_{j}}\right)}}  $$

where *SW*(.,.) is Smith Waterman score. $S_{g_{i}}$ is the information of sequence for gene *g*_*i*_.

**d) GIP kernel similarity** GIP is also employed to build gene GIP kernel $\left (K^{g}_{GIP}\right)$. Given two genes *g*_*i*_ and *g*_*j*_(*i*,*j*=1,2,...,*m*), the GIP kernel can be calculated as follows:

13 $$  K^{g}_{GIP}\left(g_{i},g_{j}\right)= \exp \left(-\gamma_{g}||\mathbf{Y}_{g_{i}}-\mathbf{Y}_{g_{j}}||^{2}\right)  $$

where $\mathbf {Y}_{g_{i}}$ and $\mathbf {Y}_{g_{j}}$ are the information of associations for vector gene *g*_*i*_ and *g*_*j*_. *γ*_*g*_ (set as 0.5) is the bandwidth of GIP kernel.

### Multiple kernel learning

In our work, two kernels in the disease space including $K^{d}_{SEM}$ and $K^{d}_{GIP}$, and four kernels of gene space including $K^{g}_{SW}, K^{g}_{GO}, K^{g}_{PPI}$ and $K^{d}_{GIP}$. We then need to combine these kernels by means of linear combination in order to achieve the optimal ones.

14 $$ \begin{aligned} \mathbf{K^{*}}=\sum_{i=1}^{k}\omega_{i}\mathbf{K}_{i}\\ \mathbf{K}_{i}\in \mathbf{R}^{N\times N}\\ \sum_{i=1}^{k}\omega_{i} = 1 \end{aligned}  $$

where *k* is the number of kernels and *ω*_*i*_ is the weight of the kernel **K**_*i*_. *N* is the number of samples in kernel **K**_*i*_.

The method CKA-MKL is utilized to combine gene kernels and disease kernels, respectively. The cosine similarity between **K**_1_ and **K**_2_ is defined as follows:

15 $$  CA\left(\mathbf{K}_{1},\mathbf{K}_{2}\right)= \frac{<\mathbf{K}_{1},\mathbf{K}_{2}>_{F}}{||\mathbf{K}_{1}||_{F}||\mathbf{K}_{2}||_{F}}  $$

where **K**_1_,**K**_2_∈**R**^*n*×*n*^,<**K**_1_,**K**_2_>_*F*_=*Trace*(**K**_1_^*T*^**K**_2_) is the Frobenius inner product and $||\mathbf {K}_{1}||_{F}=\sqrt {<\mathbf {K}_{1},\mathbf {K}_{1}>_{F}}$ is Frobenius norm.

The higher the cosine value, the greater the similarity between the kernels. CKA is based on the assumption that the combined kernel (feature space) should be similar to the ideal kernel (label space). Therefore, the alignment score between the combined kernel and the ideal kernel should be maximized. The objective function of centered kernel alignment is as follows:

16 $$ \begin{aligned} \max\limits_{\boldsymbol{\omega} \ge 0} CA\left(\mathbf{K}_{\boldsymbol{\omega}},\mathbf{K}_{ideal}\right) \\ =\max\limits_{\boldsymbol{\omega}\ge 0}\frac{<\mathbf{K}^{c}_{\boldsymbol{\omega}},\mathbf{K}_{ideal}>_{F}}{||\mathbf{K}^{c}_{\boldsymbol{\omega}}||_{F}||\mathbf{K}_{ideal}||_{F}}\\ \text{subject to} \mathbf{K}_{\boldsymbol{\omega}} = \sum_{i=1}^{k}\omega_{i}\mathbf{K}_{i}\\ \mathbf{K}^{c}_{\boldsymbol{\omega}} = \mathbf{U}_{N}\mathbf{K}_{\omega}\mathbf{U}_{N} \end{aligned}  $$

where $\sum _{i=1}^{k}\omega _{i}=1, \omega _{i} \ge 0, i=1,2,...,k$. $\mathbf {U}_{N} = \mathbf {I}_{N}-(1/N)\mathbf {l}_{N}\mathbf {l}^{T}_{N}$ denotes a centering matrix, and **I**_*N*_∈**R**^*N*×*N*^ is the N-order identity matrix, **l**_*N*_ is the N-order vector with all entries equal to one. $\mathbf {K}^{c}_{\boldsymbol {\omega }}$ is the centered kernel matrix associated with **K**_***ω***_. Equation 16 can be written as follow:

17 $$  \min\limits_{\boldsymbol{\omega} \ge 0} \boldsymbol{\omega}^{T}\mathbf{M}\boldsymbol{\omega} - 2\boldsymbol{\omega}^{T}\mathbf{a}  $$

where $\mathbf {a}\,=\,\!\left (\!<\!\!\mathbf {K}^{c}_{1},\!\mathbf {K}_{ideal}\!\!>_{F}\!,\!<\!\mathbf {K}^{c}_{2},\!\mathbf {K}_{ideal}\!\!>\!\!_{F},...\!<\!\!\mathbf {K}^{c}_{k},\!\mathbf {K}_{ideal}\!\!>\!\!_{F}\!\right)\!^{T} \!\!\in \mathbf {R^{k\times 1}}$ and **M** denotes the matrix defined by $\mathbf {M}_{ij}=<\mathbf {K}^{c}_{i},\mathbf {K}^{c}_{j}>_{F}$, for *i*,*j*=1,...,*k*. We can obtain the weight (***ω***) by solving this simple quadratic programming problem.

CKA-MKL estimates the weights of $w_{d} \in \mathbf {R}^{k_{d}\times 1},w_{g} \in \mathbf {R}^{k_{g}\times 1}$, to combine disease $\left (\mathbf {K}^{d}_{SEM},\mathbf {K}^{d}_{GIP} \in \mathbf {R}^{n\times n}\right)$ and gene $\left (\mathbf {K}^{g}_{SW},\mathbf {K}^{g}_{GO},\mathbf {K}^{g}_{PPI},\mathbf {K}^{g}_{GIP} \in \mathbf {R}^{m\times m}\right)$ kernels, separately. *k*_*d*_ and *k*_*g*_ are the number of kernels in disease space and gene space. In order to obtain the optimal kernel matrix $\mathbf {K}^{*}_{d}$ and $\mathbf {K}^{*}_{g}$ in the two spaces, first calculate the weights of kernel matrices in each space by Eq. 17, and then combine them by Eq. 14. Here, $\mathbf {K}^{d}_{ideal} = \mathbf {Y}_{train}\mathbf {Y}^{T}_{train} \in \mathbf {R}^{n\times n}$ in the disease space; and $\mathbf {K}^{g}_{ideal} = \mathbf {Y}^{T}_{train}\mathbf {Y}_{train} \in \mathbf {R}^{m\times m}$ in the gene space.

### Hypergraph learning

**Fig. 8:**
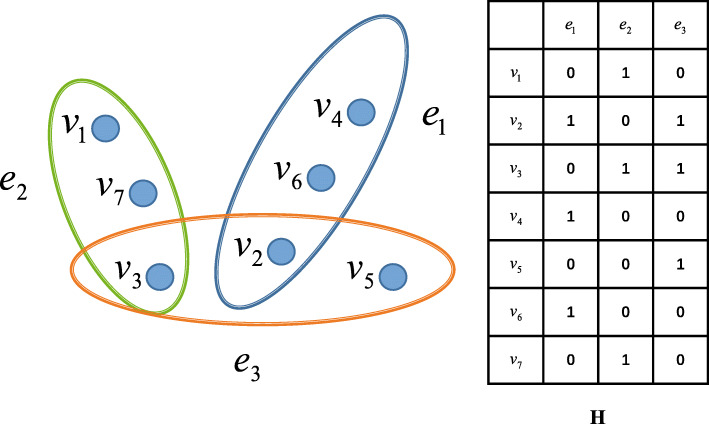
Hypergraph model in our method

In graph theory, a graph represents the pairwise relationship between a group of objects. In traditional graph structures, vertices represent objects, and edges represent relationships between two objects. However, traditional graph structures cannot express more complex relationships. For example, they cannot express more than three relationships in pairs. Hypergraph [31] solves this problem well. In hypergraph theory, this kind of multi-object relationship is represented by using a subset of vertex sets as super edges. In this study, we use hypergraph to establish this higher-order relationship. In Fig. 8 (left), {*v*_1_,*v*_2_,...,,*v*_7_} represents the vertex set, and {*v*_2_,*v*_4_,*v*_6_} are contained in hyperedge *e*_1_. Each hyperedge may comprise two or more vertices. The hyperedge will degenerate into a normal edge, when there are only two vertices in the hyperedge.

The construction of hypergraph is similar to that of ordinary graph. Hypergraph also needs a vertex set **V**, an hyperedge set **E** and the weight of hyperedge $\phantom {\dot {i}\!}\pmb {w} \in \mathbf {R}^{N_{e}\times 1}$. Here, each hyperedge *e*_*i*_(*i*=1,2,...,*N*_*e*_) is given a weight *w*(*e*_*i*_). The difference is that the hyperedge set of a hypergraph is actually a set of vertices. Therefore, a hypergraph can be represented by **G**=(**V**,**E**,*w*).

For the hypergraph **G**, the incidence matrix **H** conveys the affinity between vertices and hyperedges. And, each element of **H** can be given by the following formula:

18 $$ H(v,e)= \left\{\begin{array}{ll} 1& \text{if} v \in e \\ 0& \text{if} v \not\in e \end{array}\right.  $$

The matrix **H** describes the relationship between vertices and is shown in Fig. 2 (right). Specifically, **H**_*i*,*j*_=1 means the vertex *v*_*i*_ is included in the hyperedge *e*_*j*_. On the contrary, **H**_*i*,*j*_=0 means that the vertex *v*_*i*_ is not in the hyperedge *e*_*j*_

In a hypergraph **G**. The degree of each vertex and hyperedge and the weight of hpyperdege are expressed as follows:

19 $$ \begin{aligned} d(v) &= \sum\limits_{e \in \mathbf{E}} H(v,e)\\ \delta(e) &=\sum\limits_{v \in \mathbf{V}} H(v,e)\\ w(e_{j}) &= \sum_{i=1}^{k}K^{*}(v_{i},v_{j}) \end{aligned}  $$

where *K*^∗^ is the combined kernel.

The hypergraph is constructed using the *k* Nearest Neighbor (kNN) algorithm. Specifically, each vertex as the center point, and find the *k* vertices with the largest similarity according to the kernel matrix to form a hyperedge. Assuming that there are *N* samples, we can construct *N* hyperedges. In this study, we define the weight of each hyperedge is the sum of kernel values of the *k* vertices closest to center point, and finally the weight is normalized.

Then, we compute three matrices **D**_*v*_,**D**_*e*_ and **D**_*w*_, where **D**_*v*_ and **D**_*e*_ are the diagonal matrices of *d*(*v*) and *d*(*e*). **D**_*w*_ is the matrix of hyperedge weights.

20 $$ \begin{aligned} &\mathbf{D}_{v} = diag(d)\\ &\mathbf{D}_{e} = diag(\delta)\\ &\mathbf{D}_{w} = diag(w) \end{aligned}  $$

The hypergraph Laplacian matrix **L**^*h*^ [31] is defined as follows:

21 $$ \begin{aligned} \mathbf{L}^{h} &= \mathbf{I}-\pmb{\Theta}\\ \pmb{\Theta} &= \mathbf{D}^{-1/2}_{v}\mathbf{H}\mathbf{D}_{w}\mathbf{D}^{-1}_{e}\mathbf{H}^{T}\mathbf{D}^{-1/2}_{v} \end{aligned}  $$

where **I** is the identity matrix.

Consequently, we can obtain the hypergraph Laplacian matrix $\mathbf {L}^{h}_{d}$ and $\mathbf {L}^{h}_{g}$ about the disease and gene spaces, respectively.

### Dual hypergraph regularized least squares

Baesd on LapRLS method, we propose a novel model to predict the associations of genes and diseases, named Dual Hypergraph Regularized Least Squares (DHRLS), through incorporation of the multiple informations of gene and disease feature spaces into the dual hypergraph regularized least squares framework. The objective function can be written as follow:

22 $$ \min E(\mathbf{F}^{*}) = ||\mathbf{K}^{*}_{d}\pmb{\alpha}_{d}+\left(\mathbf{K}^{*}_{g}\pmb{\alpha}_{g}\right)^{T}-2\mathbf{Y}_{train}||^{2}_{F}  $$

where $\mathbf {F}^{*}_{d}=\mathbf {K}^{*}_{d}\pmb {\alpha }_{d}$ and $\mathbf {F}^{*}_{g}=\mathbf {K}^{*}_{g}\pmb {\alpha }_{g}$. The **F**^∗^ could be calculated by $\mathbf {F}^{*}=\left (\mathbf {F}^{*}_{d}+(\mathbf {F}^{*}_{g})^{T}\right)/2$. **F**^∗^ is an average combination of gene and disease space evaluation as the final prediction result.

Then to avoid overfitting of *α*_*d*_ and *α*_*g*_ to training data, we apply L2 (Tikhonov) regularization to Eq. 22 by adding two terms regarding *α*_*d*_ and *α*_*g*_.

23 $$ \begin{aligned} \min E(\mathbf{F}^{*}) &= ||\mathbf{K}^{*}_{d}\pmb{\alpha}_{d}+\left(\mathbf{K}^{*}_{g}\pmb{\alpha}_{g}\right)^{T}-2\mathbf{Y}_{train}||^{2}_{F}\\ &+\beta\left(||\pmb{\alpha}_{d}||^{2}_{F}+||\pmb{\alpha}_{g}||^{2}_{F}\right) \end{aligned}  $$

where *β* is a regularization coefficient.

Since previous studies [52] have shown that graph regularization terms are beneficial to improve the prediction effect of the model, graph regularization terms related to genes and diseases are added to the model. According to the local invariance assumption [53], if two data points are close in the intrinsic geometry of the data distribution, then the representations of these two points with respect to the new basis, are also close to each other. This assumption plays an essential role in the development of various kinds of algorithms. In our model, we minimize the distance between the potential feature vectors of two adjacent diseases and genes respectively

24 $$ \begin{aligned} \min\limits_{\pmb{\alpha}_{d}}\sum_{i,r}^{n}\mathbf{K}^{*}_{d}(i,r)||{\mathbf{F}^{*}_{d}}^{i}-{\mathbf{F}^{*}_{d}}^{r}||^{2}\\ =tr\left(\pmb{\alpha}^{T}_{d}\mathbf{K}^{*}_{d}\mathbf{L}_{d}\mathbf{K}^{*}_{d}\pmb{\alpha}_{d}\right)\\ \min\limits_{\pmb{\alpha}_{g}}\sum_{j,q}^{m}\mathbf{K}^{*}_{g}(j,q)||{\mathbf{F}^{*}_{g}}^{j}-{\mathbf{F}^{*}_{g}}^{q}||^{2}\\ =tr\left(\pmb{\alpha}^{T}_{g}\mathbf{K}^{*}_{g}\mathbf{L}_{g}\mathbf{K}^{*}_{g}\pmb{\alpha}_{g}\right) \end{aligned}  $$

where ${\mathbf {F}^{*}_{d}}^{i}$ is the *i*-th row vector of $\mathbf {F}^{*}_{d}=\mathbf {K}^{*}_{d}\pmb {\alpha }_{d}\in \mathbf {R}^{n\times m}, i,r=1,2,...,n$. Similarly, ${\mathbf {F}^{*}_{g}}^{j}$ is the *j*-th row vector of $\mathbf {F}^{*}_{g}=\mathbf {K}^{*}_{g}\pmb {\alpha }_{g}\in \mathbf {R}^{m\times n},j,q=1,2,...,m$. ${\mathbf {F}^{*}_{d}}^{i}$ and ${\mathbf {F}^{*}_{g}}^{j}$ mean the representations of the new base. $\mathbf {K}^{*}_{d}(i,r)$ and $\mathbf {K}^{*}_{g}(j,q)$ are the weights of two points in two spaces respectively. After adding the graph regular term, the objective function is redefined as follows:

25 $$ \begin{aligned} \min E(\mathbf{F}^{*}) &= ||\mathbf{K}^{*}_{d}\pmb{\alpha}_{d}+\left(\mathbf{K}^{*}_{g}\pmb{\alpha}_{g}\right)^{T}-2\mathbf{Y}_{train}||^{2}_{F}\\ &+\lambda_{d}tr\left(\pmb{\alpha}^{T}_{d}\mathbf{K}^{*}_{d}\mathbf{L}_{d}\mathbf{K}^{*}_{d}\pmb{\alpha}_{d}\right) \\ &+\lambda_{g}tr\left(\pmb{\alpha}^{T}_{g}\mathbf{K}^{*}_{g}\mathbf{L}_{g}\mathbf{K}^{*}_{g}\pmb{\alpha}_{g}\right)\\ &+\beta\left(||\pmb{\alpha}_{d}||^{2}_{F}+||\pmb{\alpha}_{g}||^{2}_{F}\right) \end{aligned}  $$

where *λ*_*d*_ and *λ*_*g*_ are the coefficients of graph regular terms.

We take formula 25 as a model, called Dual Graph Regularized Least Squares (DGRLS). In order to be able to express the high-order relationship between nodes, while improving the prediction effect, Hypergraph Laplacian matrix is applied to our final model DHRLS. Thus, the final objective function can be described as follows:

26 $$ \begin{aligned} \min E(\mathbf{F}^{*}) &= ||\mathbf{K}^{*}_{d}\pmb{\alpha}_{d}+\left(\mathbf{K}^{*}_{g}\pmb{\alpha}_{g}\right)^{T}-2\mathbf{Y}_{train}||^{2}_{F}\\ &+\lambda_{d}tr\left(\pmb{\alpha}^{T}_{d}\mathbf{K}^{*}_{d}\mathbf{L}^{h}_{d}\mathbf{K}^{*}_{d}\pmb{\alpha}_{d}\right)\\ &+\lambda_{g}tr\left(\pmb{\alpha}^{T}_{g}\mathbf{K}^{*}_{g}\mathbf{L}^{h}_{g}\mathbf{K}^{*}_{g}\pmb{\alpha}_{g}\right)\\ &+\beta\left(||\pmb{\alpha}_{d}||^{2}_{F}+||\pmb{\alpha}_{g}||^{2}_{F}\right) \end{aligned}  $$

where **L**^*h*^ is the hypergraph laplacian matrix, it can be calculated by Eq. 21.

### Objective function optimization for DHRLS

We select alternating least squares to estimate *α*_*d*_ and *α*_*g*_, and then run alternatingly until convergence.

**Optimizing*α***_***d***_ Suppose *α*_*g*_ are known, to obtain the optimal *α*_*d*_ by setting *∂E*(**F**^∗^)/*∂α*_*d*_=0:

27 $$ {} \begin{aligned} \frac{\partial E(\mathbf{F}^{*})}{\pmb{\alpha}_{d}}&=0\\ \mathbf{K}^{*}_{d}\left(\mathbf{K}^{*}_{d}\pmb{\alpha}_{d}+\pmb{\alpha}^{T}_{g}(\mathbf{K}^{*}_{g})^{T}-2\mathbf{Y}_{train}\right)&+\beta\pmb{\alpha}_{d}+\lambda_{d}\mathbf{K}^{*}_{d}\mathbf{L}^{h}_{d}\mathbf{K}^{*}_{d}\pmb{\alpha}_{d}=0\\ \left(\mathbf{K}^{*}_{d}\mathbf{K}^{*}_{d}+\beta\mathbf{I}+\lambda_{d}\mathbf{K}^{*}_{d}\mathbf{L}^{h}_{d}\mathbf{K}^{*}_{d}\right)\pmb{\alpha}_{d} &= 2\mathbf{K}^{*}_{d}\mathbf{Y}_{train}-\mathbf{K}^{*}_{d}\pmb{\alpha}^{T}_{g}\mathbf{K}^{*}_{g}\\ \pmb{\alpha}_{d} = \left(\mathbf{K}^{*}_{d}\mathbf{K}^{*}_{d}+\beta\mathbf{I}+\lambda_{d}\mathbf{K}^{*}_{d}\mathbf{L}^{h}_{d}\mathbf{K}^{*}_{d}\right)&^{-1}\left(2\mathbf{K}^{*}_{d}\mathbf{Y}_{train}-\mathbf{K}^{*}_{d}\pmb{\alpha}^{T}_{g}\mathbf{K}^{*}_{g}\right) \end{aligned}  $$

**Optimizing*α***_***g***_ Similarly, suppose *α*_*d*_ are known, to obtain the optimal *α*_*g*_ by setting *∂E*(**F**^∗^)/*∂α*_*g*_=0:

28 $$ {} \begin{aligned} \frac{\partial E(\mathbf{F}^{*})}{\pmb{\alpha}_{g}}&=0\\ \mathbf{K}^{*}_{g}\left(\mathbf{K}^{*}_{g}\pmb{\alpha}_{g}+\pmb{\alpha}^{T}_{d}(\mathbf{K}^{*}_{d})^{T}-2\mathbf{Y}^{T}_{train}\right)&+\beta\pmb{\alpha}_{g}+\lambda_{g}\mathbf{K}^{*}_{g}\mathbf{L}^{h}_{g}\mathbf{K}^{*}_{g}\pmb{\alpha}_{g}=0\\ \left(\mathbf{K}^{*}_{g}\mathbf{K}^{*}_{g}+\beta\mathbf{I}+\lambda_{g}\mathbf{K}^{*}_{g}\mathbf{L}^{h}_{g}\mathbf{K}^{*}_{g}\right)\pmb{\alpha}_{g} &= 2\mathbf{K}^{*}_{g}\mathbf{Y}^{T}_{train}-\mathbf{K}^{*}_{g}\pmb{\alpha}^{T}_{d}\mathbf{K}^{*}_{d}\\ \pmb{\alpha}_{g} = \left(\mathbf{K}^{*}_{g}\mathbf{K}^{*}_{g}+\beta\mathbf{I}+\lambda_{g}\mathbf{K}^{*}_{g}\mathbf{L}^{h}_{g}\mathbf{K}^{*}_{g}\right)&^{-1}\left(2\mathbf{K}^{*}_{g}\mathbf{Y}^{T}_{train}-\mathbf{K}^{*}_{g}\pmb{\alpha}^{T}_{d}\mathbf{K}^{*}_{d}\right) \end{aligned}  $$

The final prediction result is by combining the matrices in the two spaces:

29 $$  \mathbf{F}^{*}=\frac{\mathbf{K}^{*}_{d}\pmb{\alpha}_{d}+(\mathbf{K}^{*}_{g}\pmb{\alpha}_{g})^{T}}{2}  $$

The overview of our method is shown in Table 9.

**Table 9:** The algorithm of our proposed method

Algorithm : The algorithm of our proposed method
**Input:** Known associations **Y**_*train*_∈**R**^*n*×*m*^, disease space kernels ($\mathbf {K}^{d}_{SEM},\mathbf {K}^{d}_{GIP}\in \mathbf {R}^{n\times n}$) and gene space kernels ($\mathbf {K}^{g}_{GO},\mathbf {K}^{g}_{PPI},\mathbf {K}^{g}_{SW},\mathbf {K}^{g}_{GIP}\in \mathbf {R}^{m\times m}$), parameters *λ*_*d*_,*λ*_*g*_,*β* and *k*-Nearest Neighbor for DHRLS;
**Output:** Predicted associations **F**^∗^∈**R**^*n*×*m*^;
1.Calculating disease and gene kernels, listed in Table 8;
2.Calculating disease kernel weights *w*_*d*_ and gene kernel weights *w*_*g*_ by Eq. 17 (CKA-MKL), respectively;
3.Calculating $\mathbf {K}^{*}_{d}$ and $\mathbf {K}^{*}_{g}$ by Eq. 14, respectively;
4.Calculating $\mathbf {L}^{h}_{d}$ and $\mathbf {L}^{h}_{g}$ by Eq. 21, respectively;
5.Solving Eqs. 27 and 28 (ALSA), and estimating **F**^∗^ by Eq. 29;

## Data Availability

The datasets, codes and corresponding results of our model are available at https://github.com/guofei-tju/DHRLS. Associations for all genes associated with the 960 diseases are available at http://cssb2.biology.gatech.edu/knowgene. Six types of real networks are published at [44].

## Abbreviations

DHRLS: Dual Hypergraph Regularized Least Squares
DGRLS: Dual Graph Regularized Least Squares
CKA: Centered Kernel Alignment
MKL: Multiple Kernel Learning
ALSA: Alternating Least squares algorithm
MKSVM: Multiple Kernel Support Vector Machine
CV: cross-validation
AUPR: Area Under the Precision-Recall curve
AUC: Area under the receiver operating characteristic curve
GIP: Gaussian Interaction Profile
kNN: *k* Nearest Neighbor
LapRLS: Laplacian Regularized Least Squares
PPIs: Protein-protein interactions
GO: Gene Ontology
CC: cellular component
SW: Smith Waterman
